# Elevated troponin T after acute ischemic stroke: Association with severity and location of infarction

**Published:** 2015-01-05

**Authors:** Siamak Abdi, Shahram Oveis-Gharan, Farnaz Sinaei, Askar Ghorbani

**Affiliations:** Department of Neurology, School of Medicine, Shariati Hospital, Tehran University of Medical Sciences, Tehran, Iran

**Keywords:** Troponin, Stroke, Location, National Institutes of Health Stroke Scale, Electrocardiography, Creatinine

## Abstract

**Background: **Serum troponin elevation, characteristic of ischemic myocardial injury, has been observed in some acute ischemic stroke (AIS) patients. Its cause and significance are still controversial. The purpose of this study is to find determinants of troponin elevation and its relationship with stroke severity and location.

**Methods:** Between January 2013 and August 2013, 114 consecutive AIS patients confirmed by diffusion-weighted magnetic resonance imaging were recruited in this study. Serum troponin T level was measured as part of routine laboratory testing on admission. Ten lead standard electrocardiogram (ECG) was performed and stoke severity was assessed based on National Institutes of Health Stroke Scale (NIHSS).

**Results: **Troponin T was elevated in 20 (17.6%) of 114 patients. Patients with elevated troponin were more likely to have higher age, higher serum creatinine and ischemic ECG changes. Troponin levels were higher in patients with more severe stroke measured by NIHSS [7.96 (6.49-9.78) vs. 13.59 (10.28-18.00)]. There was no association between troponin and locations of stroke and atrial fibrillation. There were 6 (5%) patients with elevated troponin in the presence of normal creatinine and ECG.

**Conclusion: **Stroke severity, not its location, was associated with higher troponin levels. Abnormal troponin levels are more likely, but not exclusively, to be due to cardiac and renal causes than cerebral ones.

## Introduction

Troponin is a sensitive marker of myocardial injury.^1^ Rise in serum troponin is characteristic for myocardial ischemic injury; however it can rise in several other conditions (e.g. renal failure, heart failure, pulmonary edema, and sepsis).^2^^,^^3^ In the last decade, much interest has been drawn to the importance of serum troponin level in acute stroke. Previous studies have shown that troponin is elevated in 10-30% of acute stroke patients.^4^^-^^6^ This rise can be due to concomitant coronary artery disease and myocardial infarction (MI), congestive heart failure, renal insufficiency or direct neurogenic myocardial injury.^7^^,^^8^ Some researchers have found association between troponin level and location and size of infarction, severity of stroke [measured by National Institutes of Health Stroke Scale (NIHSS)], ischemic electrocardiogram (ECG) changes and increased mortality.^9^^-^^12^ The purpose of this study is to investigate the relationship between cardiac troponin T and severity and location of a stroke.

## Materials and Methods

Subjects with a diagnosis of acute ischemic stroke (AIS) presenting to Shariati Hospital Tehran, Iran from January 2013 to August 2013 were enrolled. Stroke patients were diagnosed according to World Health Organization definition (sudden neurological deficit that has a presumable vascular etiology) and were diagnosed as AIS if their brain computed tomography (CT) scan was normal or showed acute ischemic changes. Ischemic stroke was confirmed by showing diffusion restriction on diffusion-weighted imaging magnetic resonance imaging (MRI) using Siemens Magnetom Avanto 1.5 Tesla (Siemens Medical Solutions, Erlangen, Germany). When MRI could not be performed (e.g. cardiac pacemaker), acute stroke was confirmed by showing new hypodensity on repeat brain CT scan 4 days later. Stoke severity was assessed based on NIHSS.

Serum troponin T was measured as part of routine laboratory testing on admission. Levels of Troponin T were measured by Elecsys and cobas e analyzer (Roche diagnostics) and was considered abnormal if it was ≥ 24 ng/l. Ten lead standard ECG was performed and repeated every hour if there was any sign of ischemic changes (i.e., ST-T changes and Left Bundle Branch Block).

Descriptive statistics was shown as mean. P-P plot (which plots a variable cumulative proportion against cumulative proportions of a normal distribution, if the selected variable follows a normal distribution, points cluster around a straight line) and Kolmogorov–Smirnov test were used to assess normal distribution violation of variables. T-test and Kruskal–Wallis test, when non-parametric test had to be used, were used to compare groups in their troponin levels. Spearman correlation coefficient was used to assess the correlation between NIHSS and troponin level. Univariate general linear model was used to control for confounding variables in assessment of the association between stroke severity and troponin level. P < 0.500 was considered as significant. All analyses were done using SPSS software (version 21, SPSS Inc., Chicago, IL, USA).

## Results

A total of 120 AIS patients were enrolled. After review of patients’ records, 6 of them were excluded because their troponin levels were not measured. Compared with included patients, they were non-significantly younger (59.67 ± 17.33 vs. 66.34 ± 14.98), more masculine (83.3 vs. 55.3%), and had non-significant less severe strokes (6.17 ± 5.04 vs. 8.18 ± 6.59).

Table 1 shows patients’ basic characteristics. The precise location of a stroke could not be determined in 9 (7.9%) patients because MRI could not be done and repeated CT was inconclusive. Mean time of onset to admission was 23.76 ± 31.01 h. If 6 patients who were admitted more than three days after onset of symptoms were excluded, mean time of onset to admission would be 17.75 ± 17.85.

Table 2 shows ECG changes seen among patients. ECG ischemic changes were seen in 20% of patients.

Table 3 shows brain areas that were affected by infarcts. About two-third of strokes occurred in posterior circulation.

**Table 1:** Basic characteristics of enrolled acute ischemic stroke (AIS) patients

**Basic characteristic**	**Value**
Age (mean ± SD)	66.34 ± 14.98
Female sex [n (%)]	51 (44.7)
Time from onset (Hour) [n (%)]	
< 5	17 (14.9)
5-12	39 (34.2)
13-24	24 (21.1)
25-48	11 (9.6)
49-72	5 (4.4)
≥ 73	6 (5.3)
Unknown	12 (10.5)
NIHSS [n (%)]	
0-9	74 (64.9)
10-19	31 (27.2)
20-42	9 (7.9)
Use of rTPA [n (%)]	5 (4.4)
Abnormal serum creatinine [n (%)]	9 (7.9)
Serum troponin (ng/l)	
Minimum	1
Maximum	356
Mean ± SD	22.61 ± 43.63
Median	11.75
Abnormal [n (%)]	20 (17.5)

NIHSS: National Institutes of Health Stroke Scale, rTPA: Recombinant tissue plasminogen activator

**Table 2:** Electrocardiogram (ECG) changes of enrolled acute ischemic stroke (AIS) patients

**ECG changes**	**n (%)**
ST elevation	3 (2.5)
ST depression	6(5.0)
T inversion	16(14.2)
ST-T changes	20(16.7)
Atrial fibrillation	15(12.5)
Dynamic ECG changes	3(2.5)
LBBB	5(4.2)
RBBB	3(2.5)
Ischemic changes (ST-T, LBBB)	25(21.9)

ECG: Electrocardiogram; LBBB: Left bundle branch block; RBBB: Right bundle branch block

**Table 3:** Infarct locations of enrolled acute ischemic stroke (AIS) patients

**Location of stroke**	**n (%)**
Right hemisphere	59 (56.2)
Left hemisphere	52 (49.5)
Brainstem	21 (20.0)
Cerebellum	13 (12.4)
Frontal	52 (49.5)
Parietal	32 (30.5)
Temporal	8 (7.6)
Occipital	7 (6.7)
Insula	24 (22.9)
Basal ganglia	21 (20.0)
Internal capsule	13 (12.4)
Thalamus	5 (4.8)
Lacunar	22 (21.0)
Cortical	51 (48.6)

**Figure 1:**
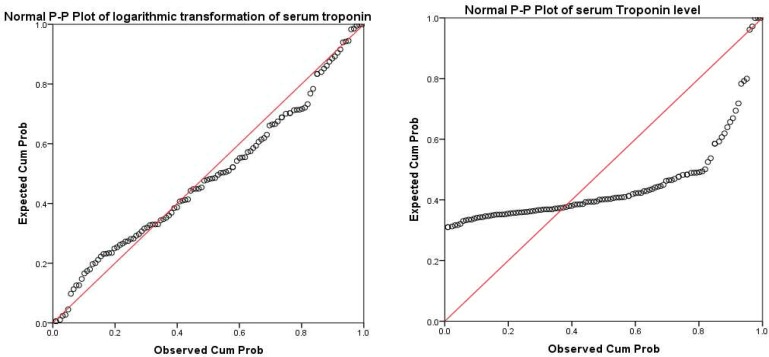
P-P plot of serum troponin level and its logarithmic transformation

Mean of serum troponin was 22.61 ± 43.63. It was abnormal in 20 (17.5%) patients. Figure 1 shows that serum troponin level did not have a normal distribution (P < 0.001). After logarithmic transformation it had a normal distribution (P = 0.260), and was used in the transformed shape in further analyses. 

Table 4 shows association of different variables with serum troponin levels in bivariate analyses. Serum troponin was significantly higher among older, male, uremic stroke patients, or patients who had ischemic changes in their ECGs.

**Figure 2:**
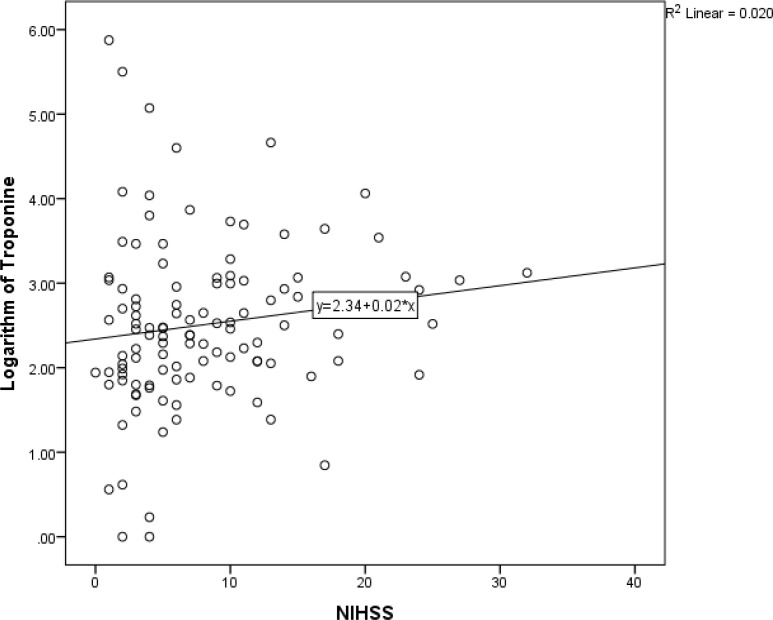
Association between National Institute of Health Stroke Scale (NIHSS) and serum troponin level among acute ischemic stroke patients

Although dichotomizing NIHSS did not yield significant association between this variable and serum troponin, figure 2 shows that there was a weak linear association between NIHSS and serum troponin (spearman correlation coefficient = 0.20; P = 0.030). Among other stroke variables, only cerebellum strokes were significantly associated with serum troponin.

Four stroke variables which had P < 0.200 in univariate analyses were entered in a multivariate analysis after exclusion of subjects who had either abnormal creatinine levels or ischemic ECG changes. Only NIHSS remained a significant predictor of troponin levels. After adjustment by age and sex, NIHSS was still a significant predictor (P = 0.002). However, the effect of NIHSS on troponin level was too small in a way that mean of troponin among stroke patients with NIHSS < 10 was 7.96 [95% confidence interval (CI) = 6.49-9.78] compared with 13.59 (95% CI = 10.28-18.00) in patients with NIHSS ≥ 10. And, among subjects with ECG ischemic signs, NIHSS was not a significant predictor of troponin (71.55 ± 112.05 vs. 29.88 ± 29.35 in subjects with NIHSS 0-9 vs. 10-42, respectively).

There were 78 AIS subjects without any evidence of renal impairment or ECG ischemic signs. 8 of them (10%) had abnormal troponin levels; 6 of them were older than 70 and 4 had NIHSS more than 9. All 8 subjects had at least one of these predictors.

## Discussion

Stroke is the second-fourth most common cause of death, after ischemic heart disease (IHD);^13^ meanwhile, IHD is the second most common cause of death after stroke.^14^^,^^15^ While stroke and IHD share the same risk factors (i.e., hypertension, hyperlipidemia, diabetes mellitus and smoking), it is not unexpected to see both diseases in one patient.^16^ Many studies have shown elevated serum troponin in significant proportion of acute stroke patients (11-36.4%).^4^^,^^17^^-^^19^

17.5% of acute stroke patients in our study had elevated serum troponin level which was congruent with previous studies. Associations, causes and value of troponin rise have been the core of many studies. It has been demonstrated that troponin elevation could be associated with higher age, more severe stroke, larger stroke, specific stroke locations, renal dysfunction, previous IHD, ECG changes and poor outcome.^20^^-^^23^ In our study, this association was found just between troponin T and age, renal impairment, ECG changes and stroke severity (i.e. NIHSS). 

We did not measure infarction size, and outcome determination was not in design of our study. Renal impairment and ischemic ECG changes were greater determinant than NIHSS; in a way that in patients with high creatinine or ischemic ECG changes, effect of NIHSS on troponin was not significant. However in patients without ischemic ECG or renal problem, higher troponin was seen in patients with more severe stroke. This effect was independent and could not be explained by other factors.

**Table 4:** Association of different variables with serum troponin levels among acute ischemic stroke (AIS) patients

**Variable**	**Troponin (ng/l)**	**Logarithm of troponin**	**P (on logarithm of troponin)**
Sex			0.008
Male	29.44 ± 56.10	2.73 ± 0.99	
Female	14.18 ± 16.44	2.24 ± 0.92	
Age			0.002
< 70	22.56 ± 56.70	2.23 ± 1.08	
≥ 70	22.67 ± 25.03	2.79 ± 0.80	
Serum creatinine (mg/dl)			0.001
≤ 1.5	20.25 ± 42.78	2.43 ± 0.94	
> 1.5	52.51 ± 49.39	3.56 ± 1.00	
Ischemic changes			< 0.001
No	14.63 ± 15.00	2.32 ± 0.86	
Yes	53.43 ± 87.44	3.16 ± 1.20	
NIHSS			0.140
0-9	24.08 ± 52.52	2.41 ± 1.08	
10-42	19.90 ± 18.46	2.70 ± 0.76	
Right hemisphere			0.760
No	17.00 ± 17.04	2.49 ± 0.86	
Yes	25.96 ± 57.75	2.44 ± 1.09	
Left hemisphere			0.250
No	23.84 ± 57.75	2.35 ± 1.06	
Yes	20.20 ± 26.12	2.57 ± 0.91	
Brainstem			0.750
No	22.43 ± 47.17	2.48 ± 0.99	
Yes	20.47 ± 34.53	2.40 ± 1.02	
Cerebellum			0.045
No	14.41 ± 14.78	2.34 ± 0.82	
Yes	76.00 ± 110.10	3.33 ± 1.58	
Insula			0.240
No	21.44 ± 47.41	2.40 ± 1.00	
Yes	24.05 ± 35.26	2.67 ± 0.93	
Frontal			0.690
No	25.57 ± 57.65	2.50 ± 1.00	
Yes	18.44 ± 25.98	2.42 ± 0.99	
Parietal			0.740
No	23.50 ± 50.52	2.44 ± 1.06	
Yes	18.71 ± 28.00	2.51 ± 0.80	
Occipital			0.410
No	22.77 ± 46.19	2.48 ± 1.00	
Yes	11.85 ± 12.03	2.16 ± 0.80	
Temporal			0.660
No	22.52 ± 46.49	2.45 ± 1.01	
Yes	16.18 ± 10.47	2.61 ± 0.63	
Thalamus			0.080
No	22.78 ± 45.76	2.50 ± 0.98	
Yes	7.20 ± 4.31	1.70 ± 1.00	
Basal ganglia			0.150
No	20.62 ± 46.38	2.39 ± 0.97	
Yes	27.75 ± 38.18	2.74 ± 1.04	
Internal capsule			0.390
No	23.49 ± 47.55	2.49 ± 1.03	
Yes	11.77 ± 10.17	2.24 ± 0.65	
Cortical			0.980
No	25.57 ± 57.16	2.46 ± 1.10	
Yes	18.30 ± 26.09	2.46 ± 0.87	

NIHSS: National Institute of Health Stroke Scale

It has been hypothesized that damage to centers regulating autonomic function can cause autonomic dysregulation resulting in sympathetic overflow and neurogenic myocardial injury.^24^ Therefore, insula and brainstem were center of attention of many researchers.^25^ Some studies have shown that troponin level is higher in the right insular, brainstem or middle cerebral artery territory infarction.^5^^,^^10^^,^^26^^,^^27^ Nevertheless, our findings along with the study of Barber and Morton. did not corroborate those results, and we found no correlation between stroke location and troponin level.^28^

Apart from associations of it, the possible causes of troponin elevation in AIS have been investigated previously. Jensen et al.^7^ and Scheitz et al.^29^ proposed that the most likely causes of increased troponin in AIS patients are silent acute MI before stroke, heart failure and renal insufficiency. Our data also show that most troponin elevations in AIS patients occurred in the context of renal or cardiac dysfunction and 3 (2.5%) of the patients had concomitant acute MI. However, there were 8 patients with elevated troponin (mean 43.75) at the presence of normal ECG and renal function. In these patients, troponin elevation could not be attributed to renal or cardiac problem, and neurogenic cardiac injury could be suspected. Nonetheless, we did not go through those cases and coronary status was not evaluated to be sure of the absence of coronary artery disease as predisposing factor for troponin elevation.

According to our findings, although there are some AIS patients with possible neurogenic myocardial injury, it is prudent to be vigilant in those with high troponin and perform appropriate cardiac and renal evaluation.

This was the first Troponin-Stroke relationship study in Iranian population. The number of patients (sample volume) was limited to 114, which made specific location stroke groups small, and might have made probable associations statistically insignificant. As mentioned, we did not evaluate cardiac structural (echocardiography) and coronary status; therefore the number of real neurogenic myocardial injury patients might have been over- or underestimated.

## Conclusion

Troponin T elevation in AIS patients was associated with higher age, creatinine, ECG changes and severity of stroke, but location of stroke was not a determinant factor. Cardiac and renal impairment were the cause of troponin elevation in the majority of patients; however, there are some patients with possible neurogenic myocardial injury.

## Conflict of Interests

The authors declare no conflict of interest in this study.

## Notes

**How to cite this article: **Abdi S, Oveis Gharan Sh, Sinaei F, Ghorbani A. Elevated troponin T after acute ischemic stroke: Association with severity and location of infarction. Iran J Neurol 2015; 14(1): 35-40.
